# A Lightweight Bioinspired SMA-Based Grasping Mechanism for Flapping Wing MAVs

**DOI:** 10.3390/biomimetics10060364

**Published:** 2025-06-04

**Authors:** Ahmad Hammad, Mehmet Süer, Sophie F. Armanini

**Affiliations:** 1 Chair of eAviation, Department of Aerospace and Geodesy, TUM School of Engineering and Design, Technical University Munich, 80333 Munich, Germany; mehmet.suer@outlook.com (M.S.); s.armanini@imperial.ac.uk (S.F.A.); 2 Department of Aeronautics, Imperial College London, London SW7 2AZ, UK

**Keywords:** gripper, shape memory alloys, FWMAVs, aerial robotics, bioinspiration

## Abstract

This study presents a novel, bioinspired perching mechanism designed to enhance the landing and takeoff capabilities of flapping wing micro aerial vehicles (FWMAVs). Drawing inspiration from the human hand, the lightweight gripper integrates a compliant claw structure actuated by shape memory alloys (SMAs) that mimic muscle movement. These SMA springs act as compact, lightweight substitutes for traditional actuators like motors or solenoids. The mechanism operates via short electrical impulses that trigger both opening and closing motions. A detailed design process was undertaken to optimize phalange lengths for cylindrical grasping and to select appropriate SMAs for reliable performance. Weighing only 50 g, the gripper leverages the high power-to-weight ratio and flexibility of SMAs, with the springs directly embedded within the phalanges to reduce size and mass while preserving high-force output. Experimental results demonstrate fast actuation and a grasping force of approximately 16 N, enabling the gripper to hold objects of varying shapes and sizes and perform perching, grasping, and carrying tasks. Compared to existing solutions, this mechanism offers a simpler, highly integrated structure with enhanced miniaturization and adaptability, making it especially suitable for low-payload MAV platforms like FWMAVs.

## 1. Introduction

The rapid advancements in aerial robotics have expanded the applications of micro aerial vehicles (MAVs), with multirotor and fixed-wing platforms being the most widely used. More recently, bioinspired aerial systems, particularly flapping wing micro aerial vehicles (FWMAVs), have gained increasing interest due to their unique advantages, such as enhanced safety (due to the absence of exposed propellers), agility, high efficiency at small scales, and low noise generation. These characteristics make FWMAVs well-suited for applications like search and rescue missions and environmental monitoring [1]. However, FWMAVs face significant limitations, including restricted payload capacity and limited flight endurance, as they cannot carry larger batteries. These constraints hinder their effectiveness in prolonged missions.

Perching has emerged as a potential solution to address the endurance limitations of MAVs. Perching mechanisms can significantly extend mission durations and expand operational flexibility by enabling vehicles to land and attach to structures such as branches or other objects. Over the past two decades, various perching techniques, including grasping, have been explored, primarily for multirotors due to their ease of integration and higher payload capacity. Fixed-wing platforms have also been investigated to a lesser extent. Examples of these mechanisms in varying weight and force ranges have been summarized in [2,3]. These designs can be broadly divided into three categories. These are grasping [4,5,6,7], embedding [8,9], and attaching-based mechanisms [10,11,12]. In contrast, perching mechanisms for FWMAVs remain underexplored despite their potential, which could enhance overall mission duration and versatility. Developing perching solutions for FWMAVs is particularly challenging due to their complex flight dynamics, limited payload capacity, and stringent weight constraints.

While significant progress has been made in perching techniques, further advancements are needed to enable practical applications, particularly for FWMAVs. The grasping mechanism is the most appropriate for FWMAVs since it provides versatility and can easily be adapted to vehicles of various weight categories without significant design alterations. Limited work has been performed for FWMAV grasping [4,13]. Most existing perching and grasping mechanisms are platform-specific [14,15,16,17]. While these mechanisms can be adapted for other platforms, such adaptations often require significant design modifications, limiting their versatility and practicality. Additionally, traditional grippers rely on rotary components, motors, and actuators, which add weight and complexity, making them unsuitable for lightweight platforms like FWMAVs. Soft robotics provides an inspiration for biomimetic designs that are adaptive and lightweight [18,19,20].

The primary objective of this work was to develop a lightweight, adaptable perching mechanism that can be seamlessly integrated across diverse MAV platforms, including fixed-wing, rotary-wing, and FWMAVs. Shape Memory Alloy (SMA) actuators emerged as a highly promising solution to meet these requirements. To address these challenges, this paper proposes a novel SMA-based gripper that eliminates the need for rotary components, motors, and actuators, resulting in a lightweight yet robust design. SMAs offer compactness, high power-to-weight ratio, and silent operation, making them ideal for integration into FWMAVs. By leveraging SMA technology, the proposed gripper aims to overcome the limitations of traditional perching mechanisms, enabling FWMAVs to perform perching maneuvers and significantly improving their mission capabilities. This multipurpose, adaptable design simplifies testing and integration across platforms, addressing the unique challenges posed by FWMAVs and expanding their practical applications. The aim was to develop a mechanism that can be applied to different MAV weight classes and different MAV configurations and then test it on the most challenging configurations, i.e., FWMAVs. This mechanism should provide reliable perching and the ability to grasp objects of different shapes, greatly enhancing MAVs’ versatility and operational capabilities. Achieving this would require a lightweight design to ensure the vehicle can take off with its payload, exert sufficient force to operate the claws effectively, and have enough displacement from the actuator to securely close around the object and maintain grasping. The design focused on circular and cylindrical perching and does not possess landing capabilities on planar surfaces.

Numerous studies on SMAs have been conducted in the last few decades [21,22,23,24,25,26,27]. This includes the design methodology, manufacturing, and applications of SMAs. SMAs have exceptional energy dissipation and recentering capabilities, enabling application in the civil and automotive industries [28,29]. Similarly, SMA can be found in various aerospace applications [30,31,32].

SMAs have not been widely explored for perching purposes. One of the few examples of FWMAVs perching was proposed by Gomez-Tamm et al. [33] utilizing SMAs as the actuating mechanism for grasping claws. They designed a tendon-driven claw and positioned SMA actuators as muscles to generate the opening and closing motion of the claw. Utilizing these alloys outside the claw mechanism led to the design of longer legs, which meant added mass. Additionally, since the SMAs were not installed on the claws, the overall control was reduced, and larger springs were used. Their work paved the way for future research on SMA-based perching mechanisms, demonstrating their practical applications and effectiveness.

In the current work, a two-finger gripping design is proposed. The goal was to develop a bio-inspired, scalable SMA-actuated perching mechanism suitable for diverse lightweight aerial platforms. Traditional motor-based systems’ complexity, bulk, and energy demands were avoided by utilizing SMAs as actuators. This innovative approach reduced the overall weight and simplified the design, making it highly adaptable for vehicles with limited payload capacity. The mechanism’s versatility ensured minimal design adjustments required for MAVs and FWMAVs integration. Flexion in fingers was achieved using tendons routed through the phalanges, inspired by the human hand [34,35]. Positioning SMA springs within the claw mechanism leads to a smaller overall size of the mechanism and a reduction in spring size. End phalanges were designed to mimic bird claws to increase friction forces on rough surfaces [36]. Furthermore, testing was conducted on an entomopter platform weighing 102 g and an ornithopter platform weighing 450 g, excluding battery and gripper. A comparison of the current gripper with some existing mechanisms is given in Table 1 based on the number of fingers, the weight of the gripping mechanism, force-to-weight ratio, and mass ratio, i.e., the percentage mass of the gripper with respect to the total mass of the vehicle. For a more detailed comparison, refer to the review paper [2].

The remainder of the article is structured as follows. Section 2 outlines the design features of the mechanism and the testing platforms. Section 3 describes the electronic components used in the study, followed by Section 4, which presents key results and analysis. Finally, Section 5 offers the concluding remarks.

## 2. System Overview

This section outlines the hardware utilized in the present work. Specifically, it introduces the ornithopter and the entomopter platforms used and enumerates their characteristics. Subsequently, the claw mechanism and other hardware components are presented, especially the SMA spring. The manufacturing process and characteristics of these components are also detailed.

### 2.1. Drone Platforms

**Figure 1:**
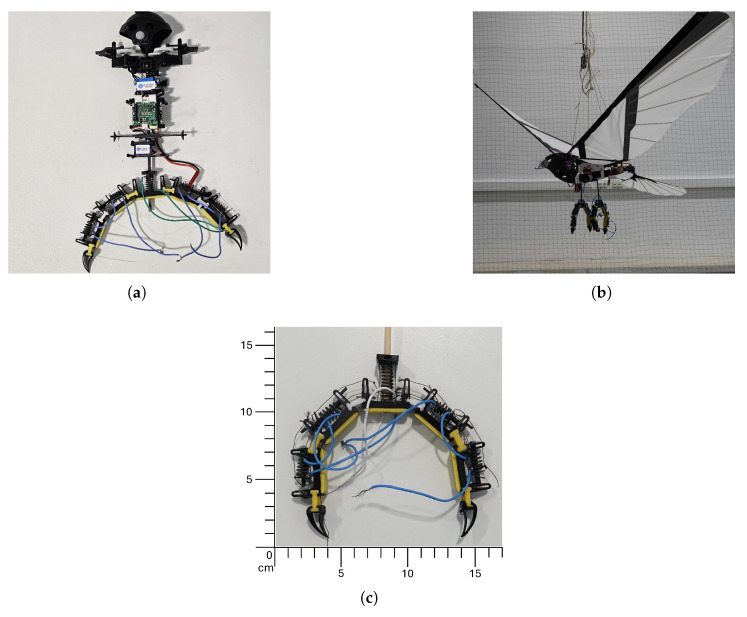
Entomopter (**a**) and Ornithopter (**b**) and claw (**c**).

The developed mechanism was tested on MAVs of different weights, both with or without hovering capability. FWMAVs were chosen as test platforms because of their posed challenge in terms of overall complexity and payload limitations. The selected platforms for testing are the VTOL-capable Nimble+ produced by Flapper Drones [42] weighing 102 g (Figure 1, left), inspired by [43] and an ornithopter platform produced by the company CarbonSail [44] weighing 450 g (Figure 1, right). Figure 1 shows both vehicles with the gripper installed. A perching mechanism suitable for the entomopter would also work on a similarly lightweight multirotor. Also, a mechanism that works on the ornithopter is expected to work on a fixed-wing MAV since they exhibit similar flight modes, e.g., entomopter and multirotor are hover-capable. In contrast, ornithopter and fixed-wing demonstrate wing-based forward flight; thus, our mechanism covers the most common MAV types, provided they can carry the mechanisms’ weight.

The primary characteristics of the entomopter are summarized in Table 2.

The physical and kinematic data of the ornithopter is given in Table 3.

In Table 2, the maximum payload capacity of the entomopter is given as 25 g. Flight tests showed the drone is capable of hovering with 65 g of payload with reduced hover time.

### 2.2. Gripper Design

**Figure 2:**
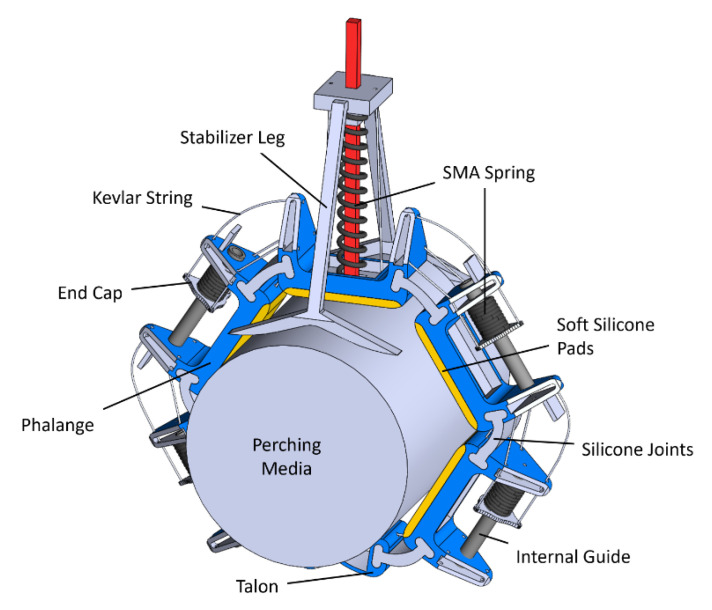
Two-finger design.

The design of the claw mechanism takes inspiration from the human hand and bird claws, but for ease and simplicity, it only uses two fingers. Forces are transferred using tendons inspired by the antagonist and protagonist muscles of human hands. This design choice decreases the weight while maintaining the perching capabilities. However, it decreases the out-of-plane stability of the finger for single claw applications, which is solved using stabilizer legs, as seen in Figure 2.

Polylactic acid (PLA) and Ecoflex 0030 two-part silicone were used to manufacture the claw. Rigid parts of the fingers were produced using PLA and 3D printing. The joints connecting the rigid parts and finger pads were made out of silicone. Utilizing soft materials adds to the elasticity of the claw and increases its compliance. Silicone, having a friction coefficient higher than PLA, was also added to the contact surface of the phalanges, fulfilling a fingertip-like function. By utilizing the elasticity of the material, the contact area and, consequently, the friction were increased further [45,46,47]. The phalanges were connected using silicone-based joints, thus giving flexibility in rotation. Each phalange had an SMA in place connected with Kevlar strings.

Taking inspiration from [35], the fingers were closed via tendons, Kevlar strings were used in our case, passing through the phalanges using SMA and opened using SMA springs located within the phalanges as shown in Figure 2. The springs would expand when a current was provided and, thus, push the end caps and pull on the Kevlar strings, resulting in the opening of the gripper. The number of phalanges was kept at three on each side to obtain the necessary deformation of SMAs to open and close the fingers and establish maximum contact with the surface. The claws could not grasp a circular cylinder properly with a two-phalange arrangement. Adding another phalange would put forth requirements for additional SMAs, thus making the setup more complicated. Roderick et al. [36] reported that the friction coefficients produced by end claws could exceed those of the toe pads by more than eight times. Consequently, a design featuring claw-shaped end phalanges (talons) was proposed to capitalize on this observed higher friction performance.

### 2.3. Modeling & Optimization

**Figure 3:**
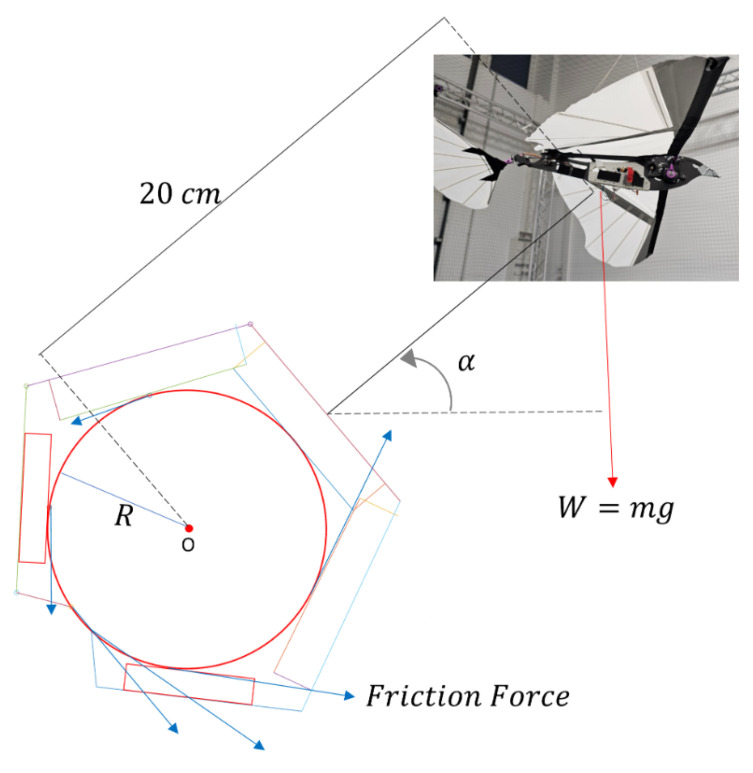
Static force balance.

The overall moment balance for the vehicle in the perched position is given as follows:

(1) $$M_{o}=M_{f}-M_{d}=0$$

(2) $$0=\left(\sum_{i=1}^{6}F_{i}\cdot R\right)-\left(W\cdot r\cdot sin\left(\alpha \right)\right)$$

where $M_{o}$ is the moment around point ‘O’ (Figure 3), $M_{f}$ is the moment due to friction forces, α is the perch angle, and $M_{d}$ is due to the drone’s weight. *F* is the friction force, *R* is the radius of the cylinder, *W* is the drone’s weight, and *r* is the distance between the center point ‘O’ and the vehicle’s center of mass.

The weight of the ornithopter platform and the payload capacity of the entomopter platform were considered while optimizing the design. The force requirements and geometric bounds were defined based on the ornithopter. However, the weight constraint was based on the payload capacity of the entomopter. It was assumed that if the design is light enough to be carried on the entomopter, it will be easily integrated on the ornithopter. Similarly, if the mechanism can bear the forces for the ornithopter, it would be able to demonstrate the same for the entomopter. For simplification, the mechanism was constrained to only perch on circular and cylindrical media. Based on the total mass with onboard equipment as 600 g, 3 cm perch radius, $75^{\circ }$ perching angle, which is the approach angle between the vehicle and the branch, and 20 cm leg length from the perching medium center and the drone’s center of gravity to avoid collision of wings with the ground during flapping, the total friction force needed to ensure moment balance was calculated as:

(3) $$\sum_{i=1}^{\infty }F_{i}=10.15\;N$$

The estimated coefficient of friction is 0.5 (averaging the friction for silicone and wood), and the total force required for a successful perch is 20.3 N. The force needed for each claw comes to 10.15 N. Additionally, the entomopter platform’s payload capacity of 50 g was kept in mind.

**Figure 4:**
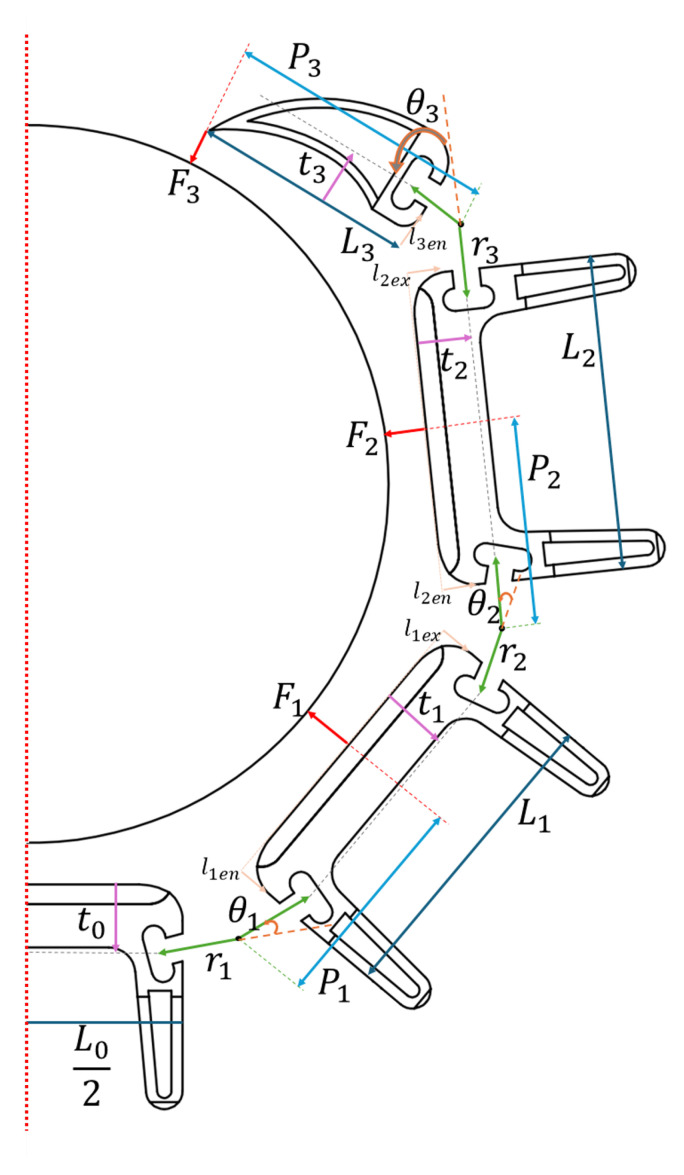
Finger parameters for one-half of the claw.

From Figure 3, it can be seen that six forces are acting on the structure due to friction. A two-dimensional force balance is considered for calculations. Since the left and right digits of the claw are symmetrical (red dotted line in Figure 4), for ease of calculations, it is assumed that $F_{1}$ = $F_{6}$, $F_{2}$ = $F_{5}$, and $F_{3}$ = $F_{4}$. One-half of the claw can be seen in Figure 4. The force calculations are performed based on the geometric and mathematical model by Dong et al. [48] and is given as:

(4) $$\begin{array}{c} F_{1}=\frac{1}{P_{1}}\left(F_{a}r_{1}-F_{2}\left(P_{2}+L_{1}cos\theta_{2}\right)-F_{3}\left(P_{3}+L_{2}cos\theta_{3}+L_{1}cos\left(\theta_{2}+\theta_{3}\right)\right)\right) \end{array}$$

(5) $$F_{2}=\frac{1}{P_{2}}\left(F_{a}r_{2}-F_{3}\left(P_{3}+L_{2}cos\theta_{3}\right)\right)$$

(6) $$F_{3}=\frac{1}{P_{3}}\left(F_{a}r_{3}\right)$$

Here, *P* is the distance between the center of the tendon to the point of force for the respective phalange, θ is the angle between two phalanges, and $F_{a}$ is the tendon force.

The design parameters were optimized to achieve the maximum force transmission to phalanges. The parameters to optimize were the phalange lengths (*L*), joint radii (*r*), phalange thicknesses (*t*), and tendon in and out positions to the phalanges (*l*). For ease of construction and calculation, the following constraints were applied.

(7) $$L_{0}=L_{1}=L_{2}$$

(8) $$t_{0}=t_{1}=t_{2}$$

(9) $$r_{1}=r_{2}=r_{3}$$

The minimum tendon force for one finger was kept to be 10 N. The minimum total force was set to 35 N per claw based on the calculations made in Equations (1)–(3) as per the configuration shown in Figure 3, where the arrows represent the friction force of each phalange.

The goal was for the claw to exert maximum force while perched and be able to grasp cylinders with 3 to 5 cm radius while keeping the overall size of the gripper small. A table containing constraints on the geometry was generated and summarized in Table 4, a Matlab script calculating the force output for every possible combination was run.

The following optimization function (10) was defined and maximized to choose the best-performing parameter set.

(10) $$F_{opt}=F_{T}+\left(4\cdot F_{t}\right)-F_{std.dev}$$

where $F_{T}$ is the total contact forces applied by the phalanges, $F_{std.dev}$ is the standard deviation of contact forces of each phalange, and $F_{t}$ is the contact force of the talon. As mentioned, the force exerted by the end effectors can be up to eight times that of the phalanges [36], depending on the conditions. It is more realistic to use a conservative value of four. The best-performing parameters set was found as follows.

- Phalanges $L_{0-2}=35$ mm and for Claw $L_{3}=25$ mm
- Thicknesses of Phalanges $t_{j}=7$ mm
- Joint radii $r_{j}=6$ mm
- Tendon entry(en) and exit(ex) points $l_{1en}=3$ mm, $l_{1ex}=4$ mm, $l_{2en}=4$ mm, $l_{2ex}=4$ mm, $l_{3en}=3$ mm

The force needed to be generated by individual phalanges was calculated. Force needed for each phalange should be $F_{1}=0.92$ N, $F_{2}=3.79$ N, and for end effector $F_{3}=3.73$ N. The total force would be the sum of all forces needed on each phalange, i.e., $\sum_{1}^{6}F_{i}$. Therefore, the selected design parameters represent a gripper that can theoretically exert a force of 16.88 N.

The bending stiffness was kept as low as possible. Following optimization, rigid components were fabricated using 3D printing with tough PLA filament, ensuring durability and precision. The choice to use PLA vs TPU came down to the high rigidity PLA provides as opposed to TPU. The joints and fingertips were manufactured using silicone, ensuring compatibility and accuracy and enabling quicker grasping capabilities. An internal guide was provided to prevent out-of-plane motion and buckling of the SMA springs.

**Figure 5:**
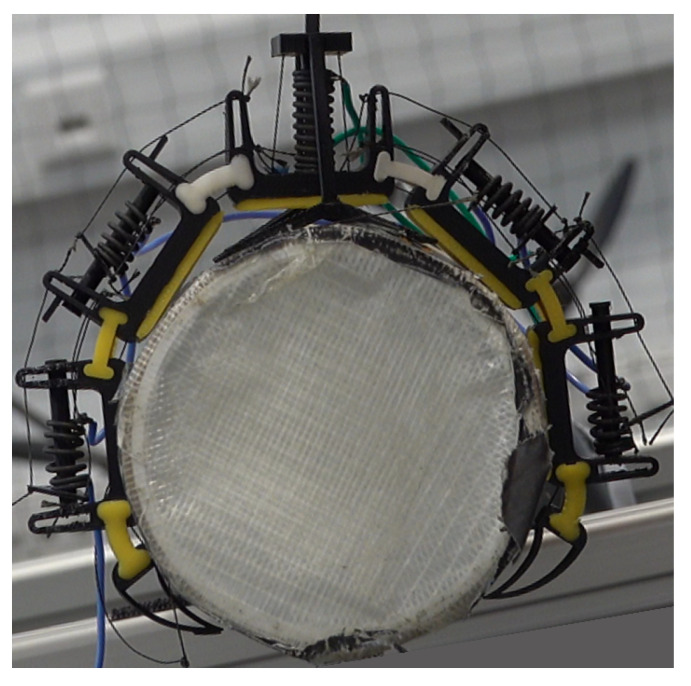
Assembled claw on a perching media.

**Figure 6:**
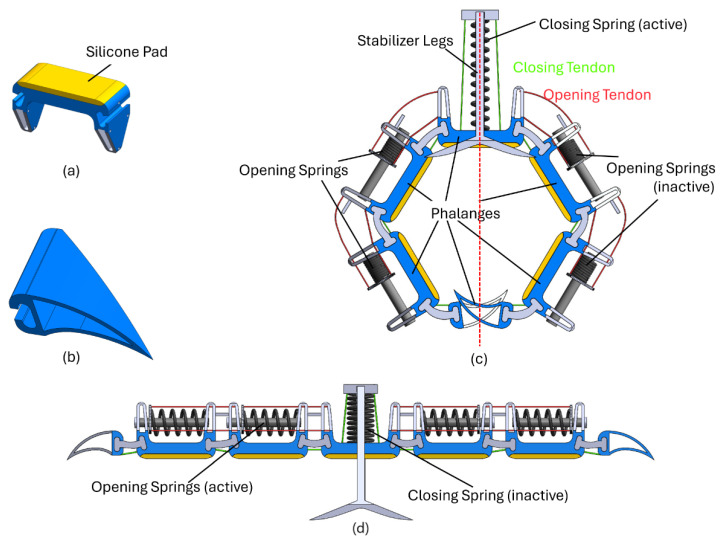
Mechanism schematic showing (**a**) phalange, (**b**) talon, (**c**) closed state of the claw, and (**d**) open state of the claw.

**Figure 7:**
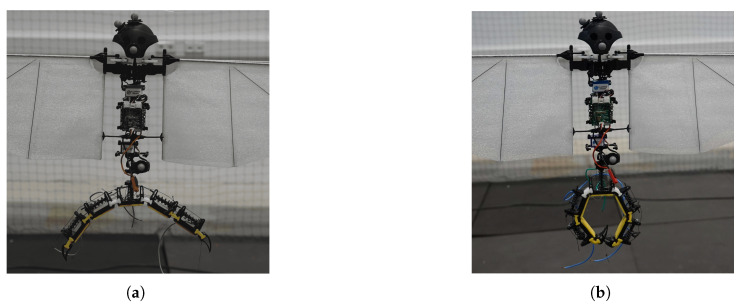
Gripper mechanism in open (**a**) and closed (**b**) state installed on FWMAV.

In total, three SMA springs were used for both the opening and closing of the claw. The parameters of these springs are given in Table 5. One spring is responsible for the closing actuation, whereas two springs, half of each for a particular phalange, are responsible for opening the claw. Finally, a high-strength, heat-resistant Kevlar string was used as tendons. The parts were assembled, and the assembled mechanism is shown in Figure 5. Figure 6 shows the actuation process. When current is provided to the closing spring, it expands, resulting in compression of the opening springs and, thus, closing the claw. By applying current to the opening springs, their expansion results in compression of the closing spring, resulting in the opening of the claw. The opening and closing position of the claw while installed on the entomopter can be seen in Figure 7.

## 3. Electronics and Control

The electronics and the control scheme implemented to activate the SMA springs are discussed in this chapter, along with their justification. The onboard battery was used in the entomopter platform due to weight restrictions. In contrast, the ornithopter platform used an external battery, which enabled quicker actuation but added to the vehicle’s overall weight. The onboard battery can also actuate the SMAs on the ornithopter platform. However, this will deplete the battery and adversely affect the vehicle’s endurance.

### 3.1. Electronic Components

The activation and deactivation of the springs were performed through a microcontroller. Some flight controllers have free channels that can be utilized to control the actuation. On MAVs with no channel available, an external microcontroller should be used.

**Figure 8:**
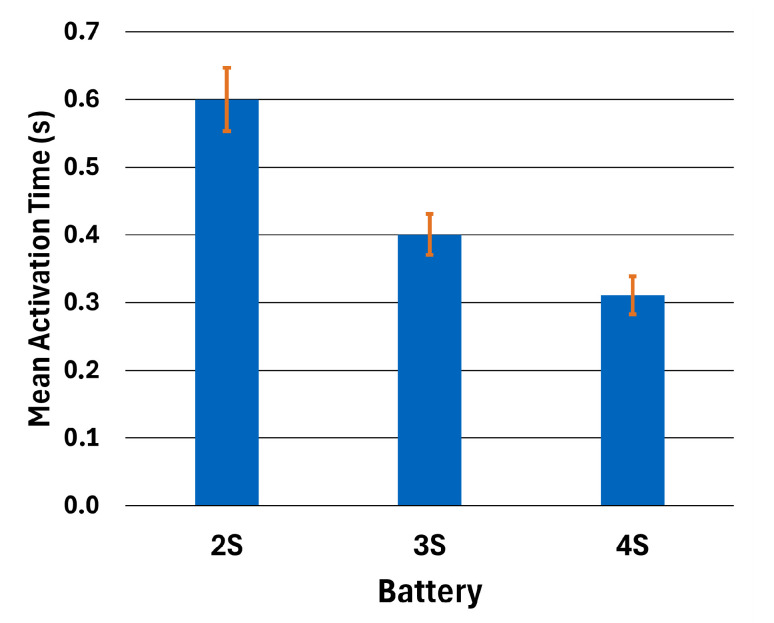
Battery activation time and standard deviation.

The SMA springs provided by Ingpuls GmbH were activated by 20 A current with a 4.3 V power supply in 1.1 s. However, experiments showed that the resistance of one full-length spring was 0.5 Ohms, including cabling. Activation experiments are repeated 10 times for each battery. The mean activation time data and standard deviation σ of one full-length spring using different batteries, without adding external resistance, are given in Table 6. A summary of comparison can be seen in Figure 8.

Experimental data showed that the activation times are lower with increasing battery voltage. However, the weight of the battery increased as a consequence. Hence, the decision was made to use the onboard battery for the entomopter platform and an external battery for the ornithopter platform.

### 3.2. Control System

**Figure 9:**
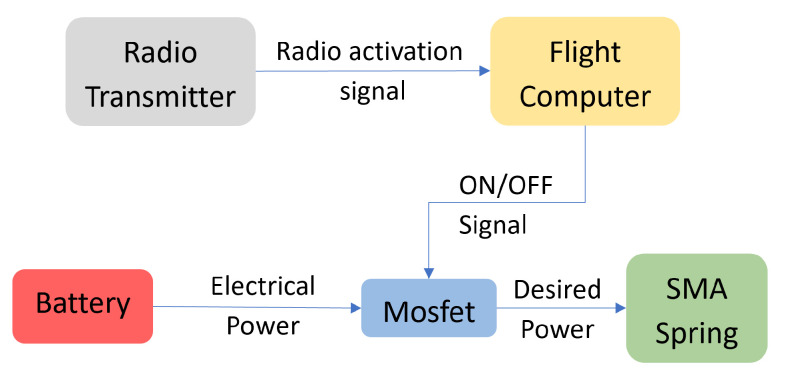
Control Scheme.

Actuation is controlled by a MOSFET used as a simple switch, and the control signal is sent from the transmitter and received by the onboard computer. When the radio input to activate the actuator arrives, a signal is sent to the respective MOSFET, and the current passes through the springs, heating and activating the actuator. The entomopter uses a Crazyflie Bolt 1.1, whereas the ornithopter has a Matek F405-VTOL flight controller onboard programmed using the INAV configurator. Tghe overall control scheme can be seen in Figure 9.

The software is designed to activate only one set of springs, either opener or closer, at a time and for a predetermined duration. This characteristic prevents accidental activation of antagonist springs and controls the temperature to prevent parts from getting damaged due to the high temperatures the springs can reach. Thus, the current is provided for a short duration, therefore preventing the springs from reaching higher temperatures and also not wasting additional energy by supplying continuous energy. An activation signal is sent to the onboard computer, and an impulse signal lasting one second is supplied to the spring.

Experiments [33] show that the strength of SMA depends on the temperature. After cooling down, they try and return to their original state. Therefore, additional current should be provided to the springs at specific intervals to maintain their strength and ensure successful perch. The duration of intervals to provide the current has been investigated through experiments and discussed in the next chapter.

## 4. Results and Discussion

**Figure 10:**
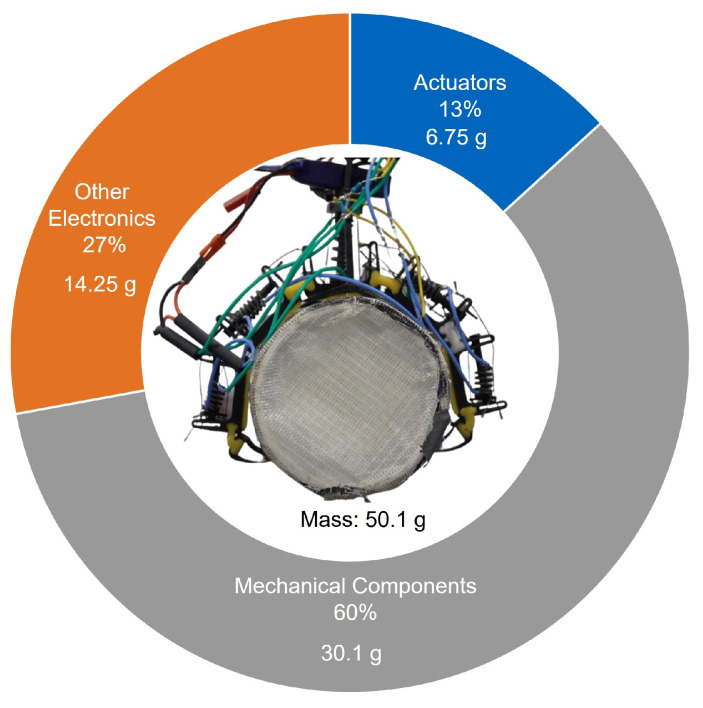
Mass distribution of the gripper mechanism.

The complete prototype of the gripper mechanism was obtained by integrating all the mentioned components. The final prototype weighs 50 g, including all electronic and mechanical components, excluding the battery. The existing battery used to power the MAV can be used as the power source for the gripper, or, depending on the mission requirements, an external battery can be added. The gripper is suitable for a wide range of small to medium MAVs. Figure 10 shows the mass distribution of the mechanism. Compared with similar works [13,49], the actuator mass is less than that of traditional servo motors. However, complex design requirements to accommodate unconventional actuators and extra electronic components bring the mass up. Utilizing smarter design choices can reduce the mass of the gripper, making it suitable for use in MAVs with even lower weight ranges.

The force generated by individual phalanges was experimentally measured using load cells to validate compliance with the design requirements. The cells are placed on a cylindrical surface such that the force is perpendicular to the load cell. Each force is measured individually. The measured forces were as follows: $F_{1+6}=1.23$ N, $F_{2+5}=6.27$ N, and $F_{3+4}=8.46$ N for both end effectors. The cumulative force exerted by the gripper was calculated to be 15.96 N. Although this value is slightly lower than the theoretically predicted force, it remains within the acceptable range and satisfies the design requirements. These results confirm the effectiveness of the gripper design and its ability to perform as intended.

Two different types of tests were conducted to validate the design of the gripper. Firstly, manual testing of the gripper was performed to show it can support the drone’s weight on specific perching media, with and without some deviation from perfect landing, e.g., angular perching, and its ability to grasp and carry different objects. Secondly, flight tests were performed to assess the flight characteristics of the drone while the gripper mechanism was attached. The tests are discussed in the following sections.

### 4.1. Static Testing of the Gripper Mechanism

The gripper’s effectiveness was validated before the control system was integrated into the drones. The gripper hand was assembled onto the entomopter platform, and the platform was manually placed on different perching media. Actuation was triggered manually using an external battery. Different geometries, dimensions, and surface textures were used in testing. Perches included three tree branches with diameters Ø40 mm, Ø60 mm, and Ø70 mm, respectively, and a circular cardboard pipe with diameter Ø82 mm. Grasps included a circular low-density polyethylene (LDPE) pipe with a diameter of Ø50 mm, a rectangular cardboard box with a cross-section of 40 mm × 40 mm, and a rectangular sponge with a cross-section of 100 mm × 40 mm.

**Figure 11:**
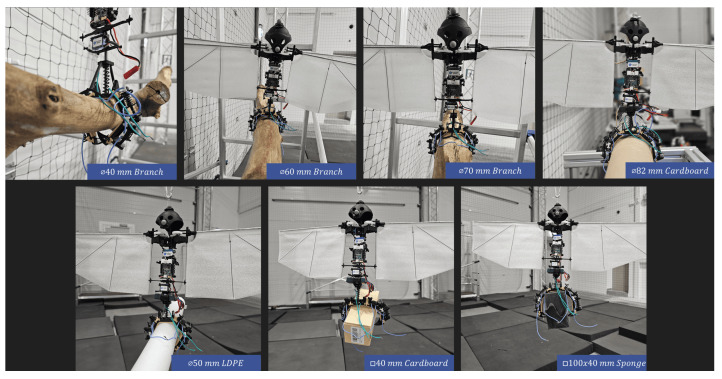
Mechanism perched on and grasping different objects.

**Figure 12:**
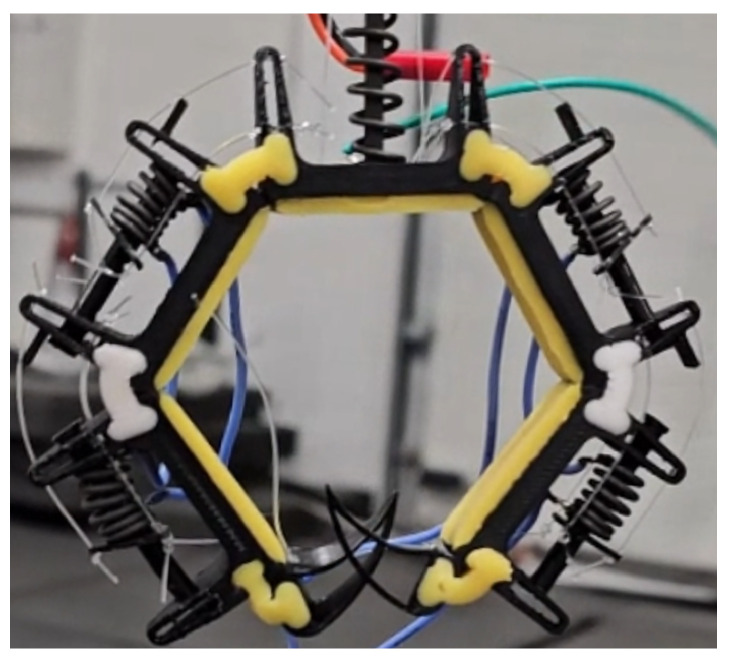
End phalanges colliding together, determining the minimum grasp radius.

The mechanism can grasp objects of different sizes within the design range as shown in Figure 11. The summary of success of perching on and grasping different media is summarized in Table 7 and Table 8, respectively. It is noted that the mechanism showed successful grasping on branches having a diameter greater than 4 cm and could also successfully grasp objects of different shapes. With lower radius objects; however, the phalanges clash with each other, and the necessary radius to fully grasp the object cannot be achieved as depicted in Figure 12.

The grasping force of the mechanism significantly decreased as the SMA springs cooled after the initial actuation. Once the initial current was applied, the power supply was cut, allowing the springs to cool and lose their gripping force. This resulted in a reduced grasping force of approximately 2 N per phalange, which was considerably lower than the force generated when the springs were heated. Consequently, the vehicle began to slip from the perch as the grip weakened. To maintain a continuous perch, energy must be supplied to the SMAs at specific intervals to reheat the springs and sustain their actuation. The relationship between the perching angle and perching duration was analyzed and is presented in Table 9. For prolonged perch durations, the current must be reapplied within the defined interval to prevent slippage.

Static testing was performed for a single gripper to demonstrate holding capacity. Various objects were grasped, and the gripper was capable of holding 25 g of mass for 20 s. Therefore, another current impulse must be given within this interval to ensure holding capacity. For the entomopter, due to weight restriction, flight is not possible with additional payload; however, the ornithopter demonstrated successful flight after carrying a 25 g additional payload while providing a current impulse after every 15 s.

As seen in Table 9, the successful perching duration depends on the perching angle. The perch duration showed significant variance due to the design of the end claws. The perch angle can be minimized in a free-flight environment with an accurate control architecture. Considering a maximum $10^{\circ }$ of a perching angle, providing a current impulse every 60 s is adequate, considering increased cooling due to higher wing gusts in an open environment.

**Figure 13:**
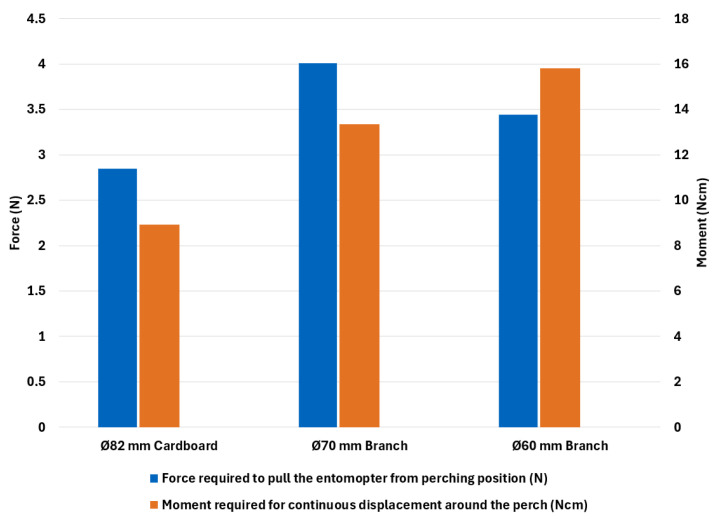
Force required to pull the entomopter from perching position (blue) & Moment required for continuous displacement around the perch (orange).

Figure 13 shows the forces (in blue) required to pull the mechanism straight upwards from a perched position, right after the perching maneuver was executed, and no continuous current impulse was given. The force needed to pull the gripper while perched at $0^{\circ }$ varies with the perch diameter since the talons are designed and optimized for a particular diameter. The embedding success of the talons increases the force required to pull the gripper.

Figure 13 also shows the moment (in orange) required for continuous displacement around the perch. The moment required also varies from perch to perch for the same reason stated above.

### 4.2. Flight Testing

**Figure 14:**
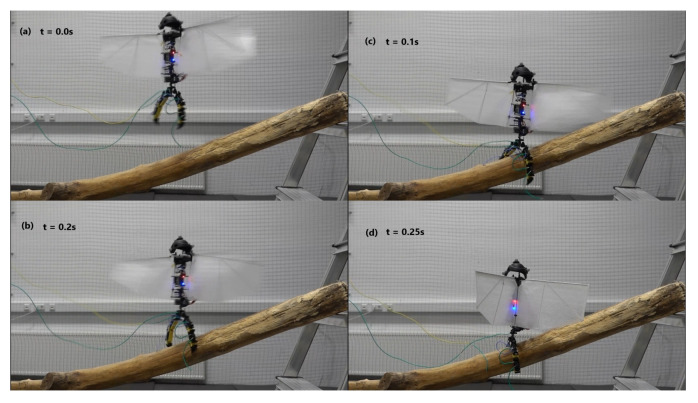
Perching test of entomopter.

**Figure 15:**
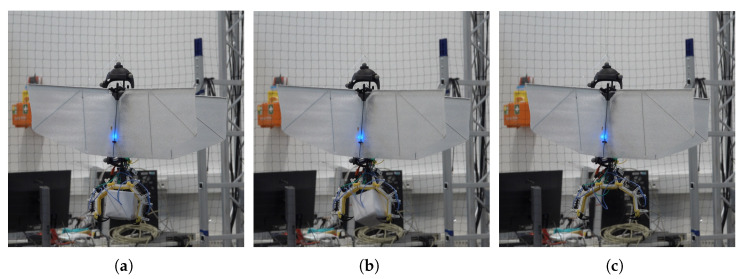
Grasp and drop a non-circular object (**a**) object grasped, (**b**) release actuation initiated, and (**c**) object dropped.

**Figure 16:**
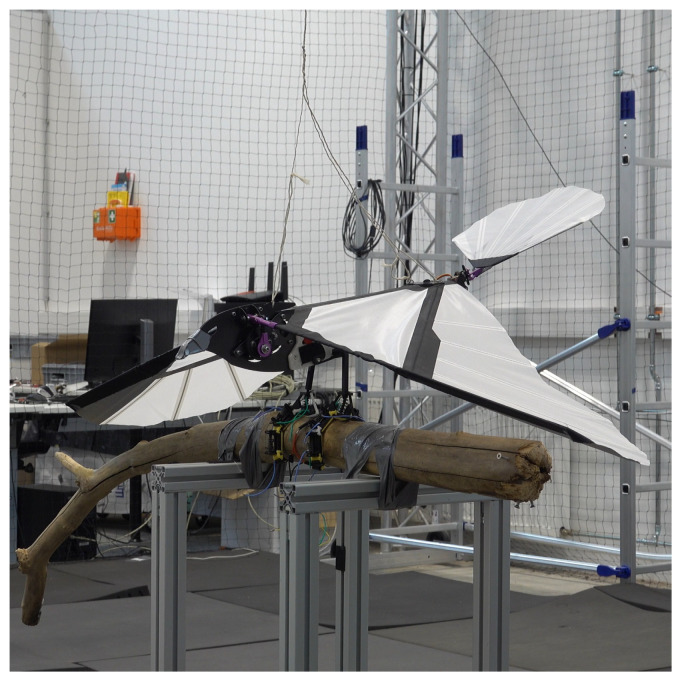
Perching test of Ornithopter.

**Figure 17:**
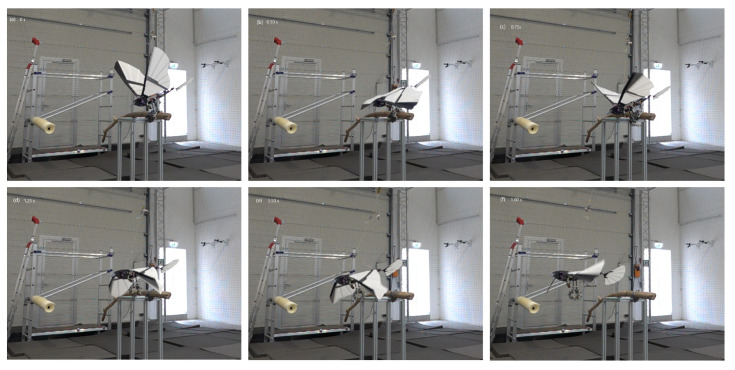
Take-off Sequence for Ornithopter.

Flight tests were conducted to evaluate flight and take-off from a perched position and demonstrate the practicability of SMAs as actuators assisting in perching. Successful take-off experiments were performed on the vehicles with the mechanism attached. The tests were repeated 15 times for the entomopter, demonstrating 100% success for takeoff. Takeoff for the ornithopter was performed 5 times with 100% success. Landing of the entomopter on a branch was also successfully achieved with 87% success (Figure 14). Landing for the ornithopter was unsuccessful. Hover duration decreased by approximately 1 min for the entomopter with 2 perching attempts, i.e, 3.5 min. While hovering with a perching mechanism, the hover time was reduced to 4.5 min. The mechanism’s ability to grasp and release objects with non-circular shapes is also demonstrated with the drop test, as shown in Figure 15. The mechanism installed on the ornithopter can be seen in Figure 16. Successful take-off for the ornithopter from a perched position was demonstrated (Figure 17). However, the ornithopter platform’s flight dynamics and control need to be improved to land successfully.

These tests showed that a successful take-off and subsequent flight are possible. The mechanism enables the entomopter and the ornithopter to perch in a realistic free-flight environment and support the weight of these platforms. With small perching angles, prolonged perch times without extra energy expense can be achieved.

## 5. Conclusions

This paper presents the design and development process of a 50 g SMA-actuated perching mechanism to be used on micro air vehicles (MAVs). We have shown that without using traditional actuation methods such as servo motors, it is possible to design actuators suitable for high-force generation that can perform numerous tasks. While having capabilities similar to traditional actuation methods, the actuator design presented in this paper is lighter than most alternatives.

The gripper mechanism presented in this paper was designed to be modular, easy to manufacture, compliant, and lightweight. Its ability to manipulate objects and to perch on different surfaces was shown experimentally. The implementation of SMA springs allows for the design of small, lightweight, and modular gripper systems capable of generating forces high enough for perching tasks, yet still light enough to be integrated into small MAVs. Successful testing on both a hover-capable entomopter and a cruise-flight capable ornithopter suggests that the design can easily be adapted to other small to medium-range MAVs, including multirotor and fixed-wing designs.

This design can be improved further. Its final state lacks repeatable actuation cycles due to the high friction forces between parts. Investigating approaches to reduce friction between the parts would allow for repeatable actuation. Another drawback of the current design is its inability to land on a planar surface. Furthermore, by sourcing a different spring, phalange lengths can be shortened, and the number of phalanges can be increased, further improving the compliance of the mechanism. The design of the individual phalanges can be optimized further, and the printing material can be changed to decrease the weight of the mechanism. These suggested improvements have the potential to further refine the design, making it more efficient and optimized for performance.

## Figures and Tables

**Table 1:** Comparison of some existing solutions.

Gripper Model	Fingers	Gripper Weight (N)	Force/ Weight	Mass Ratio	Maximum Grasped Object Size (cm)
Robot Hand [7]	3	5.41	10.36	66%	15
Adaptive [37]	2	2.91	0.20	-	12
Magnetic [38]	-	2.89	8.80	38%	21
Compliant [39]	3	0.09	6.80	25%	-
Bi-stable [40]	2	0.13	4.7	33%	3.3
Passive [41]	2	0.28	22.13	5%	-
Soft Gripper [4]	2	0.40	42.27	23%	5
**SMA**	2	0.49	32.6	8%	10

**Table 2:** Physical and kinematic data of entomopter [42].

Parameters	Values
Wingspan	0.049 m
Weight	102 g (min. take-off weight)
Payload Capacity	25 g
Flight Time	8 min (forward 3 m/s, min. weight) 5 min (hover, max. payload)

**Table 3:** Physical and kinematic data of ornithopter.

Parameters	Values
Mean chord length of each wing	0.455 m
Aspect ratio of each wing	1.42
Mass of the ornithopter	450 g
Flight speed of the ornithopter	10–25 km/h
Range of wingbeat frequency	3.5–4.5 Hz
Payload capacity	450 g

**Table 4:** Constraints for force calculation.

Parameters	Values
Phalanges	2.5–5 cm
Talons	1.5–3 cm
Thicknesses of Phalanges	3–10 mm
Joint radii	3–10 mm
Tendon entry and exit points	2–6 mm

**Table 5:** SMA spring actuator data.

Parameters	Values
Type	Pressure Spring
Wire Diameter	1.26 mm
Number of Coils	12
Block length	17 mm ± 2 mm
High Temperature	50 mm ± 8 mm

**Table 6:** Activation data for one spring using different batteries.

	2S	3S	4S
Mean Activation Time (s)	0.6	0.4	0.3
Activation Time Standard Deviation (σ)	8%	8%	9%
Voltage (V)	8.32	11.54	16.01
Current (A)	16.64	23.08	32.02
Power (W)	138.44	266.34	512.64
Activation Power (mAh)	2.78	2.56	2.66

**Table 7:** Perching attempts.

Object	Success
Branch Ø30 mm	Unsuccessful
Branch Ø40 mm	Successful
Branch Ø60 mm	Successful
Branch Ø70 mm	Successful
Cardboard Cylinder Ø82 mm	Successful

**Table 8:** Successful grasping attempts.

Object	Mass (g)	Success
LDPE Ø50 mm	17	Successful
Cardboard □ 40 × 40 mm	25	Successful
Sponge □ 100 × 40 mm	12	Successful

**Table 9:** Perch duration.

Perch Angle	Perch Duration
$5^{\circ }$	240 s
$10^{\circ }$	120 s
$15^{\circ }$	60 s
$20^{\circ }$	20 s
$180^{\circ }$ (Hang)	120 s

## Data Availability

The original contributions presented in this study are included in the article material. Further inquiries can be directed to the corresponding author.
